# “Designing synthetic topological matter with atoms and lights”

**DOI:** 10.1038/s41377-022-00738-3

**Published:** 2022-02-28

**Authors:** Entong Zhao, Chengdong He, Gyu-Boong Jo

**Affiliations:** 1 Department of Physics, The Hong Kong University of Science and Technology, Clear Water Bay, Kowloon, Hong Kong, China; 2 IAS Center for Quantum Technologies, Clear Water Bay, Kowloon, Hong Kong, China

**Keywords:** Quantum optics, Atom optics

## Abstract

One of the most interesting directions in quantum simulations with ultracold atoms is the expansion of our capability to investigate exotic topological matter. Using sophisticated atom-light couplings in an atomic system, scientists have demonstrated several iconic lattice models that exhibit non-trivial band topology in a controlled manner.

## Abstract (web summary)

With atom-light couplings in atomic systems, scientists have demonstrated several iconic lattice models that exhibit non-trivial band topology in controlled manners, which expands our capability to investigate exotic topological matter.

## Graphical abstract

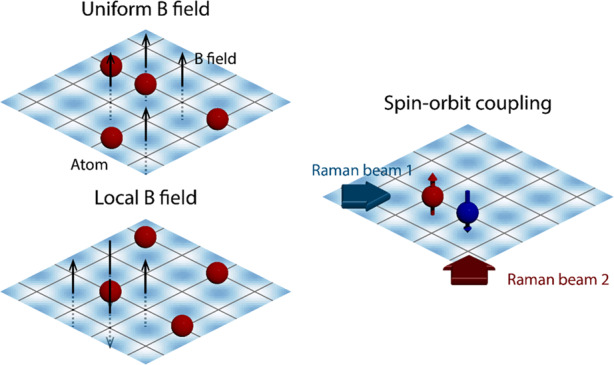

We have witnessed an ever-increasing demand for new quantum matter that will potentially revolutionize modern technologies. However, our understanding of such cutting-edge quantum matter is often impeded by the complexities related to the interactions, non-trivial topology, or dissipation in the system. This challenge has motivated the engineering of synthetic quantum systems through which a key theoretical idea can be coherently simulated and tested in an ideal manner. Among many candidates, ultracold atoms have been identified as synthetic quantum materials whose system properties can be freely tuned on demand. This feature offers a great opportunity to discover new phases of quantum matter, expand our understanding of their underlying physics, and improve the unique properties of quantum materials.

Recently, there has been a growing number of studies engineering synthetic crystalline structure containing a collection of cold atoms, which experience periodic potential induced by laser lights. In this synthetic crystal, so-called optical lattices^1^, researchers have put a lot of efforts into implementing non-trivial band topology for atoms in optical lattices, which allows to simulate topological matter. This type of matter usually possesses excitations with exotic properties—such as carrying fractional charges and obeying non-Abelian statistics- and has topologically protected boundary states. These properties may transform topological matter into a new platform for developing low-energy-dissipation electronic devices and fault-tolerant quantum computation.

One of the simplest ways to make the crystalline structure topological is to introduce magnetic fields—more generally gauge fields—on the periodic crystalline structure. Then, charged particles in solids are governed by two length scales, the lattices constant of the periodic potential and the magnetic length set by the cyclotron motion of charged particle in the magnetic field. In such a situation, band structure often exhibits a Bloch wavefunction with non-trivial topology. For the simple two-dimensional square lattice, this model, also known as the Harper-Hofstadter model, represents the simplest quantum Hall effect in lattices. Although the model was proposed more than 60 years ago, it was only recently that researchers simulated the Harper–Hofstadter lattice Hamiltonian^2,3^. It is because neutral atoms are typically charge-neutral, and therefore the synthetic gauge fields—for example, magnetic fields—need to be externally created for atoms. Researchers engineer the phase factor associated with the tunnelling process by use of laser-assisted tunnelling, and create a uniform magnetic field^2,3^ as illustrated in Fig. 1. Alternatively, non-trivial band topology can also emerge when locally staggered magnetic fields are present in a so-called Chern insulator (see Fig. 1). Haldane model, one of such iconic models, was realized by engineering the nearest and next-nearest tunnellings in a graphene-type optical lattice^4^.

**Fig. 1:**
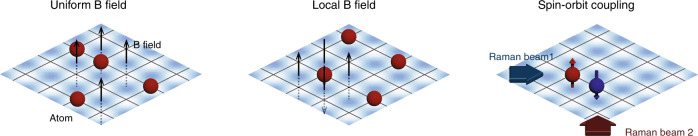
Topological lattice models with artificial gauge fields. With a uniform magnetic field generated by a pair of far-detuned laser beams, the Harper–Hofstadter model for neutral particles can be created in the optical lattice. Alternatively, one can apply locally staggered magnetic fields for realizing non-trivial band topology. Spin–orbit coupling can be implemented for a spin-1/2 system with the Raman transition resulting in topological models

Another type of important mechanism that exhibits non-trivial band topology is spin–orbit coupling, a form of gauge potential that entangles the spin and momentum of a particle. In contrast to the model Hamiltonian that describes the motion of charged particles, the spin–orbit coupling has been extensively studied in various topological matter including, but not limited to, topological insulators and anomalous Hall states. Although cold atoms do not have intrinsic spin–orbit coupling, an atomic spin—referring to the hyperfine level—can be effectively entangled with its momentum through the coupling between atomic spin states and external fields.

Researchers first demonstrated laser-induced spin–orbit coupling in bulk atomic systems by Raman coupling two spin states in a momentum-sensitive way^5^. This progress has enabled a number of groups to implement spin–orbit coupling in engineered optical lattices where Raman transitions are added^6^. In those lattices, the spin degree of freedom exhibits non-trivial structure of the Bloch band, showing spin texture in the momentum space. Several iconic topological models have been observed including the symmetry-protected topological phase^7^ and the quantum anomalous models^8^. A combination of tailored spin–orbit coupling and lattice potentials is of significant benefit in designing distinct topological band structure in high dimensions. For example, nodal-line semimetal^9^ and Weyl point^10^ have been demonstrated in 3D, which added a new recipe to quantum simulations with cold atoms.

Alternatively, artificial gauge fields or spin–orbit coupling can be implemented in a synthetic dimension together with optical lattices^11,12^. The concept of synthetic dimensions has recently emerged as a compelling approach to engineer various Hamiltonians in lattices. This approach takes advantage of suitable degrees of freedom in a low-dimensional atomic system to synthesize a higher-dimensional lattice Hamiltonian including synthetic dimensions.

Most of the concepts in topological matter have been initially proposed and realized in the solid-state systems. In these systems, most topological materials are accompanied by weakly interacting systems, where the correlation effects among electrons can be treated via mean-field type approximation or density functional theory. However, the topological matters are not limited in these weakly correlated materials. Strongly correlated systems may also have fruitful topological states, thereby driving the strong research interest in atomic systems that can tune interactions in a controlled manner. In the future, researchers examine how a synthetic topological system made of cold atoms can simulate topological states in the presence of interactions and/or dissipation. Another promising area of research will be the observation of distinct topological band structure in higher dimensions. In such a system, a high-dimensional spin–orbit coupling corresponds non-Abelian gauge potential exhibiting non-zero Berry curvature. This exciting prospect is surely worthy of further attention from the community.
